# Ofatumumab and Early Immunological Cells Subset Characterization in Naïve Relapsing Multiple Sclerosis Patients: A Real-World Study

**DOI:** 10.2174/1570159X21666230803161825

**Published:** 2023-09-25

**Authors:** Emanuele D’Amico, Aurora Zanghì, Roberta Fantozzi, Diego Centonze, Carlo Avolio

**Affiliations:** 1 Department of Medical and Surgical Sciences, University of Foggia, Foggia, Italy;; 2 IRCCS Neuromed, Pozzilli, Italy

**Keywords:** Ofatumumab, b-cells, immunophenotype, multiple sclerosis, immunological cells subset, anti-CD20

## Abstract

**Background:**

Ofatumumab (OFA) is a fully human anti-CD20 monoclonal antibody administered with a 20 mg subcutaneous monthly dosing regimen.

**Methods:**

Inclusion criteria were patients: 1) aged 18-55; 2) with a confirmed diagnosis of relapsing Multiple Sclerosis (RMS), per the revised 2010 McDonald criteria; 2) who started OFA according to Italian Medicines Agency prescription rules and within 12 months from the RMS diagnosis; 3) naïve to any disease-modifying therapy. The primary outcome was to offer an overview of cellular subsets of RMS naïve patients (time 0) and then after 4 weeks (time 1) and 12 weeks (time 2) on therapy with OFA in a real-world setting.

**Results:**

Fifteen patients were enrolled. CD3+ T cell frequencies were higher at time 1 (%80.4, SD 7.7) and time 2 (%82.6, SD 5.8) when compared to time 0 (%72.4, SD 9.8), *p* = .013. B naïve cells were barely detectable in the OFA group at time 1 (%0.4, SD 0.5) and 2 (%1.4, SD 2.9) when compared to time 0 (%11.5, SD 3.8), *p* < .001.

**Conclusion:**

The progressive and increasing use of anti-CD20 drugs imposes the need for larger, prospective, real-world, long-term studies to characterize further immunophenotypes of patients with RMS treated with OFA.

## 1 INTRODUCTION

Ofatumumab (OFA) is a fully human anti-CD20 monoclonal antibody administered with a 20 mg subcutaneous monthly dosing regimen [1-3]. It was approved for the treatment of active forms of relapsing multiple sclerosis (RMS), and it was launched on the Italian market at the end of April 2022.

OFA presents the peculiar ability to bind to two distinct regions within the large and small extracellular loops of the anti-CD20 antigen (the other anti-CD20 drugs, such as rituximab and ocrelizumab, only bind to the large one), and that determines B cell lysis through complement-dependent cytotoxicity and antibody-dependent cell-mediated cytotoxicity [2, 4, 5]. Consequently, OFA has higher potency compared with the earlier anti-CD20 drugs, depleting B cells more efficiently.

In this Italian real-world multicenter study, we aimed to describe the frequency of immunological cell subsets in a cohort of naïve RMS who started OFA, monitoring it after 4 and 12 weeks from the first administration.

## 2 METHODS

### 2.1 Setting

A real-world observational study was performed at the two MS centers of the University of Foggia and IRCSS-Neuromed, Isernia, Italy. Patients were consecutively admitted between April 1, 2022, and June 30, 2022. Data were extracted on September 30, 2022.

### 2.2 Participants

Inclusion criteria were patients: 1) aged 18-55; 2) with a confirmed diagnosis of RMS, per the revised 2010 McDonald criteria [6]; 2) who started OFA according to Italian Medicines Agency prescription rules and within 12 months from the RMS diagnosis; 3) naïve to any disease-modifying therapy (DMT).

Patients who had received immunosuppressive or immunomodulant therapies for other concomitant diseases were excluded.

### 2.3 Procedures

Demographical (age, gender, smoking status, body mass index-BMI, comorbidities) and clinical data (disease duration, disability assessed by the Expanded Disability Status Scale and number of relapses in the year before diagnosis) were collected. Baseline neuroradiological data (magnetic resonance imaging, MRI, within 3 months from diagnosis) were obtained using a 3-Tesla scanner (Magnetom-Skyra, Siemens).

Blood samples were collected in EDTA vials and processed within 2 hours. Flow cytometry acquisition was managed with the NAVIOS (Beckman Coulter). For the evaluation of B cells, we used the Dura Clone IM B Cells kit.

We identified CD16+CD56+ CD3- (Natural Killer) cells. Among CD3+ T cells, we identified the overall count of CD3+ T cells and the subsets: CD3+CD4+ (T-Helper) and CD3+CD8+ (T-Cytotoxic). After gating on the CD19-positive cells, we considered CD19+CD27−IgD+ (B-naïve) cells. The local Ethics Committee approved the study, and informed consent was obtained from all patients.

### 2.4 Statistical Analyses

Data are presented as proportion for categorical variables and mean (standard deviation) or median (interquartile range) for continuous variables. Lymphocyte subsets were compared with Anova-test with Welch correction. The linear regression model was built for ancillary analysis (reported as standardized-β-coefficient, R^2^-statistics and 95% confidence interval-CI). A *p*-value ≤ 0.05 was considered significant. Analyses were performed using SPSS 21.0 (IBM^©^, Armonk, NY, USA).

### 2.5 Outcomes

The primary outcome was to offer an overview of cellular subsets of RMS naïve patients (time 0) and then after 4 weeks (time 1) and 12 weeks (time 2) on therapy with OFA.

Ancillary, we aimed to investigate, if any, the association between statistically significant cellular subsets (time 2) and BMI.

## 3 RESULTS

**Fig. (1):**
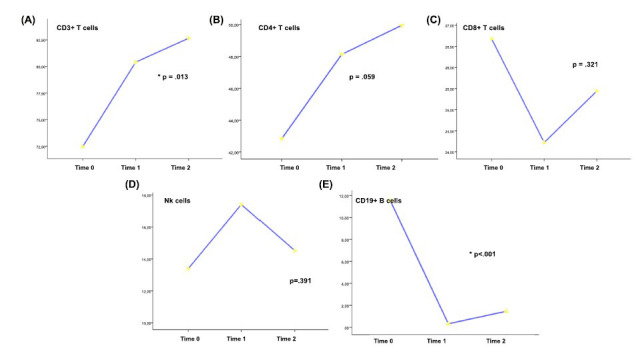
Cellular subsets frequencies during the investigated time points. **Time 0** = Baseline; **Time 1** = 4 weeks; **Time 2** = 12 weeks. **A =** TCD3+; **B =** T helper CD3+ CD4+; **C =** T cytotoxic CD3+ CD8+; **D =** Natural killer CD16+CD56+ CD3-; **E** = CD19+ B cells (B-naïve). *statistically significant by Anova with Welch correction.

A total cohort of 15 patients was enrolled. The demographic, clinical and radiological characteristics and baseline values of cellular subsets are presented in Table **1**. CD3+ T cell frequencies were higher at time 1 (%80.4, SD 7.7) and 2 (%82.6, SD 5.8) when compared to time 0 (%72.4, SD 9.8), *p*=.013 (Fig. **1A**). Likewise, we did not detect major differences in the frequencies of T-helper and T-cytotoxic cells, although there was a slight increase in T-helper cell frequencies and a corresponding slight decrease in T-cytotoxic cells frequencies from time 0 to time 2 (Figs. **1B**, **C**).

Natural Killer cells were also not significantly different during the investigated time points (Fig. **1D**).

B naïve cells were barely detectable in the OFA group at time 1 (%0.4, SD 0.5) and 2 (%1.4, SD 2.9) when compared to time 0 (%11.5, SD 3.8), *p* < .001 (Fig. **1E**).

**Fig. (2):**
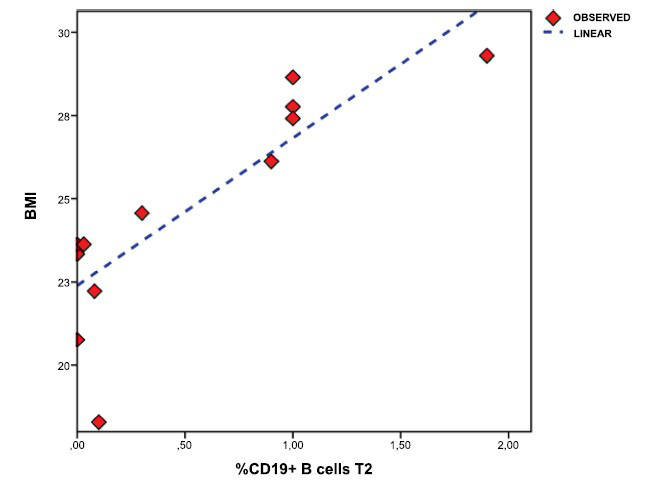
Linear regression analysis plot between CD19+ B cells (B-naïve) (time 2) and BMI values. BMI, body mass index.

The linear regression analyses revealed increased B-naïve cells cells repopulation rate with increased BMI (standardized-β-coefficient = .842, CI 95% .092-.228 *p* = .022, R^2^ = .683) (Fig. **2**).

## 4 DISCUSSION

In the present study, we first reported in a real-world setting the effect of the anti-CD20 antibody therapy OFA on peripheral immune cell populations in a cohort of RMS patients not previously exposed to any DMT.

During three months from the first infusion, OFA was effective on B lymphocytes, maintaining such an effect for the last time of observation, and we also suppose that OFA could also impact on T-lymphocytes subset.

Furthermore, patients with higher BMI had higher levels of B-naïve cells after 12 weeks.

A recent case-control study reported that a mean treatment duration of three months in the OFA group (n=10 patients) led to near complete B cell depletion in line with an altered composition of certain CD4+ T cell subpopulations, such as enhanced frequencies of naive and a decrease in non-suppressive regulatory T cells. However, the patients enrolled in this study had been treated with prior DMTs, which might have influenced the immune cell composition [7].

Moreover, it remains unknown how early OFA treatment might impact immune cell subsets.

The role of BMI in B cell repopulation in patients treated with anti-CD20 drugs has yet to be discussed. In a previous Italian study on ocrelizumab-treated MS patients, a fast repopulation rate was observed in patients with higher BMI [8].

The main strength of the study is that the immune cell subsets were reported in naïve RMS patients who were homogeneous for disease characteristics; additionally, it was conducted within the clinical practice, using the equipment available in the laboratories of the participating centers.

The main limitations of our study are the lack of more detailed analyses on lymphocyte subpopulation and their functional changes after treatment (*e.g*., cytokines), the relatively small sample size and the absence of robust longitudinal data let us to a cautious interpretation of reported data. Larger, prospective, real-world, long-term studies are needed to characterize further immunophenotypes of patients with RMS treated with OFA.

## Figures and Tables

**Table 1:** Baseline characteristics and cellular subsets of the enrolled cohort.

**-**	**RMS (n = 15)**
Female n (%)	7 (46.6)
Age (year), (mean ± SD)	30.7 ± 5.6
Smokers n (%)	4 (26.6)
Comorbidities n (%)	3 (20)
BMI (median, IQR)	23.6 (22.7-27.5)
Disease duration (mean ± SD)	8.2 ± 4.5
Relapses in the year before diagnosis (mean ± SD)	2.6 ± 0.4
Baseline EDSS (median, IQR)	1.0 (1.0-2.0)
N. of Brain MRI lesions on T2 weighted sequences (mean ± SD)	15.5 ± 9.1
N. of Brain MRI lesions on T1 gad+ weighted sequences (mean ± SD)	1.1 ± 0.2
Lymphocytes, % (SD)	-
Natural Killer CD16+CD56+ CD3-	13.4(5.4)
T CD3+	80.4(7.7)
T-helper CD3+CD4+	42.8(8.9)
T-cytotoxic CD3+CD8+	29.4(7.7)
CD4+/CD8+ (ratio, mean ± SD)	1.8 ± 0.8
B-naïve cells	11.5(3.8)

**Abbreviations**: EDSS, Expanded Disability Status Scale; MRI, magnetic resonance imaging; N.,number; SD, standard deviation.

## Data Availability

Anonymised data will be shared upon request from any qualified investigator for the sole purpose of replicating procedures and results presented in the report, provided that data transfer is in agreement with EU legislation on the general data protection regulation.

## LIST OF ABBREVIATIONS

BMI: Body Mass Index
DMT: Disease-modifying Therapy
MRI: Magnetic Resonance Imaging
OFA: Ofatumumab
RMS: Relapsing Multiple Sclerosis

## References

[1] https://www.ema.europa.eu/en/documents/product-information/kesimpta-epar-product-information_en.pdf.

[2] Kang C., Blair H.A. (2022). Ofatumumab: A review in relapsing forms of multiple sclerosis.. Drugs.

[3] Hauser S.L., Bar-Or A., Cohen J.A., Comi G., Correale J., Coyle P.K., Cross A.H., de Seze J., Leppert D., Montalban X., Selmaj K., Wiendl H., Kerloeguen C., Willi R., Li B., Kakarieka A., Tomic D., Goodyear A., Pingili R., Häring D.A., Ramanathan K., Merschhemke M., Kappos L. (2020). Ofatumumab versus teriflunomide in multiple sclerosis.. N. Engl. J. Med..

[4] D’Amico E., Zanghì A., Gastaldi M., Patti F., Zappia M., Franciotta D. (2019). Placing CD20-targeted B cell depletion in multiple sclerosis therapeutic scenario: Present and future perspectives.. Autoimmun. Rev..

[5] Sanford M., McCormack P.L. (2010). Ofatumumab.. Drugs.

[6] Thompson A.J., Baranzini S.E., Geurts J., Hemmer B., Ciccarelli O. (2018). Multiple sclerosis.. Lancet.

[7] Faissner S., Heitmann N., Plaza-Sirvent C., Trendelenburg P., Ceylan U., Motte J., Bessen C., Urlaub D., Watzl C., Overheu O., Reinacher-Schick A., Hellwig K., Pfaender S., Schmitz I., Gold R. (2022). Immune response in ofatumumab treated multiple sclerosis patients after SARS-CoV-2 vaccination.. Front. Immunol..

[8] Signoriello E., Lus G., Bonavita S., Lanzillo R., Saccà F., Landi D., Frau J., Baroncini D., Zaffaroni M., Maniscalco G.T., Curti E., Sartori A., Cepparulo S., Marfia G.A., Nicoletti C.G., Carotenuto A., Nociti V., Caleri F., Sormani M.P., Signori A. (2022). Switch from sequestering to anti-CD20 depleting treatment: Disease activity outcomes during wash-out and in the first 6 months of ocrelizumab therapy.. Mult. Scler..

